# Infectious SARS-CoV-2 is rarely present in the nasopharynx samples collected from Swedish hospitalized critically ill COVID-19 patients

**DOI:** 10.1007/s11845-022-02961-8

**Published:** 2022-03-07

**Authors:** Jiaxin Ling, Rachel A. Hickman, Robert Frithiof, Michael Hultström, Josef D. Järhult, Åke Lundkvist, Miklos Lipcsey

**Affiliations:** 1 Department of Medical Biochemistry and Microbiology, Zoonosis Science Center, Uppsala University, Uppsala, Sweden; 2 Department of Surgical Sciences, Anesthesia and Intensive Care Medicine, Uppsala University, Uppsala, Sweden; 3 Integrative Physiology, Department of Medical Cell Biology, Uppsala University, Uppsala, Sweden; 4 Department of Medical Sciences, Zoonosis Science Center, Uppsala University, Uppsala, Sweden; 5 Hedenstierna Laboratory, Department of Surgical Sciences, Anesthesiology and Intensive Care, CIRRUS, Uppsala University, Uppsala, Sweden

The coronavirus disease 2019 (COVID-19) pandemic is still rapidly spreading. It is still unclear to what extent patients with COVID-19 treated at the intensive care unit (ICU) shed infectious severe acute respiratory syndrome coronavirus 2 (SARS-CoV-2). Yet, this has important implications for infection control and disease management as well as for the understanding of the pathobiology of COVID-19 at the ICU [1]. Consequently, in a prospective study, we isolated SARS-CoV-2 from nasopharynx (NPH) samples from COVID-19 patients admitted to ICU with severe respiratory failure at the Uppsala University Hospital, between December 2020 and June 2021 (including the second half of the second wave, and the third wave). The study was approved by the National Ethical Review Agency (EPM; No. 2020–05,730). Informed consent was obtained from each patient, or next of kin if the patient was unable to give consent. The Declaration of Helsinki and its subsequent revisions were followed.

In brief, NPH samples were collected consequently from the patients twice a week during the first 2 weeks at the ICU, and then once a week until discharge from the ICU or death. We collected 199 NPH samples from 124 patients. The NPH samples were immediately stored in 2 mL of viral transfer media (HBSS supplemented with 2% FBS, 100 µg/mL gentamicin, and 0.5 µg/mL amphotericin B) and delivered on ice to the Zoonosis Science Center the same day [2]. A total of 280μL of each sample was used for a viral qRT-PCR test based on the N and E genes of SARS-CoV-2, and 100 μL was used for virus isolation in our biosafety level 3 laboratory, as described previously [3]. Infected cells were checked for development of cytopathic effect (CPE) as compared to uninfected control cells for at least two passages. Once CPE was observed, the supernatant was collected and put in TRIzol® (Thermo Fisher, USA) for inactivation and further analysis by qRT-PCR as described earlier [4, 5].

We isolated four SARS-CoV-2 strains from four individual patients (3.2% of the cohort) as confirmed by both CPE development in Vero E6 cells and by a qPCR cycle threshold (Ct) value below 20. For data analysis, we used the lower of the two Ct values obtained by the qPCRs targeting the N and E genes, respectively, for each sample. These four culturable samples had a lower Ct median values of 22.5 (interquartile range (IQR), 21–25; Fig. 1) as compared to 32.5 (IQR, 27–36) (*p* < 0.01) for the non-culturable NPH samples, which was in line with other studies [6–8]. According to the recommendation by the Centers for Disease Control and Prevention, transmission-based precautions should be taken for non-immunocompromised patients for at least 10 days from the first positive test [9]. In a previous report, 17.8% (23/129) of high-dependency or ICU patients had culturable SARS-CoV-2 virus at some time-point, and it was approximated that less than 5% have culturable virus 15 days after onset of symptoms [10]. Our findings of culturable virus in 4/124 ICU patients at least 10 days after the onset of the symptoms were similar or lower than previously reported, possibly explained by differences in case mix and the sampling time during the disease course.

No differences in duration of COVID-19, demography, previous medical history, or organ function were found between the group of patients with culturable infection and the group of patients with non-culturable infections (Table 1). However, blood hemoglobin levels and red blood cell counts on ICU admission were lower (*p* < 0.01) among patients with culturable virus potentially resulting from SARS-CoV-2-induced hemoglobin denaturation, and aggravating hypoxemia [11]. We acknowledge that the study was limited by the low statistical power given the low number of cases with culturable SARS-CoV-2.

**Table 1:** Patient characteristics in patients with and without positive virus cultures of SARS-CoV-2. Data presented as median (IQR) unless otherwise stated

	Virus could not be cultured (*n* = 120)	Virus could be cultured (*n* = 4)
Female *n* (%)	37 (31)	1 (25)
Age (yrs)	64 (54–71)	74 (58–78)
Body weight	92 (82–105)	81 (72–102)
BMI (kg/cm^2^)	30 (27–35)	26 (24–34)
*Previous medical history*		
Pulmonary disease	29 (24)	-
Hypertension	67 (56)	2 (50)
Ischemic heart disease	12 (10)	-
Diabetes mellitus	31 (25)	2 (50)
*Organ support in the ICU*		
Renal replacement therapy	2 (2)	-
Invasive ventilation	66 (55)	1 (25)
PaO_2_/FiO_2_ ratio on admission	17.3 (15.9–20.7)	18 (12.4–20.2)
Lowest PaO_2_/FiO_2_ ratio during ICU stay	11.0 (8.7–12.8)	10.6 (8.7–13.8)
* Vital signs on admission to the ICU*		
Breathing rate (/min)	28 (22–33)	23 (10–29)
Heart rate (/min)	85 (75–96)	67 (65–105)
Mean arterial pressure (mmHg)	90 (78–103)	97 (71–102)
Body temperature (°C)	37.4 (36.9–37.9)	37.8 (37.6–37.9)
SAPS3	53 (47–58)	37 (37–37)
Days after symptoms onset	10 (9–12)	10 (7–26)
***Laboratory values on ICU admission***		
Blood Hemoglobin (g/L)	132 (121–144)	110 (96–126)*
Plasma CRP (mg/L)	129 (65–200)	132 (50–259)
Plasma Procalcitonin (µg/L)	0.3 (0.1–0.6)	0.1 (0.1–7.9)
Blood WBC (× 10^9^)	7.6 (5.6–10.6)	8.7 (5.7–11.8)
Blood RBC (× 10^12^)	4.5 (4.1–4.8)	3.9 (3.3–4.2)*
Blood Platelets (× 10^9^)	245 (182–292)	197 (136–314)
Plasma Creatinine (µmol/L)	73 (61–93)	72 (70–446)
***Maximal laboratory values during ICU stay***		
Blood Hemoglobin (g/L)	145 (132–154)	125 (111–139)
Plasma CRP (mg/L)	195 (130–275)	224 (156–297)
Plasma Procalcitonin (µg/L)	0.5 (0.2–1)	7.3 (0.4–15)
Blood WBC (× 10^9^)	14.2 (11–17.7)	16 (10.2–20.5)
Blood RBC (× 10^12^)	4.7 (4.3–5)	4.2 (3.5–4.3)*
Blood Platelets (× 10^9^)	444 (355–513)	353 (230–588)
Plasma Creatinine (µmol/L)	80 (68–105)	91 (73–588)

*BMI* body mass index, *CRP* C-reactive protein, *COVID-19* coronavirus disease 2019, *ICU* intensive care unit, *SAPS3* Simplified Acute Physiology Score 3, *WBC* white blood cell count, *RBC* red blood cell count

*p < 0.05

In conclusion, we found 4 of 124 patients were shedding infectious virus up to 26 days after symptom onset, suggesting that SARS-CoV-2 is rarely culturable from the nasopharynx in hospitalized critically ill COVID-19 patients. However, we could not identify specific clinical characteristics except the hemoglobin level and the red blood cell counts identifying patients with culturable SARS-CoV-2.

**Fig. 1:**
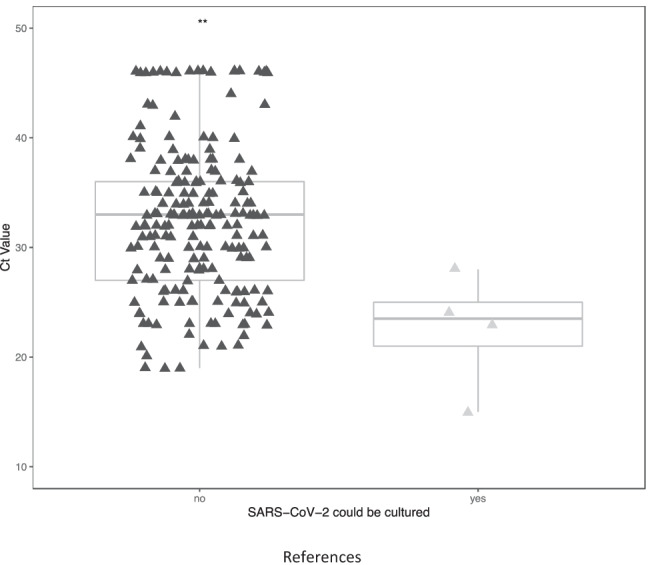
qRT-PCR data in patients with and without positive virus cultures of SARS-CoV-2. The lower Ct value from N gene- and E gene-based qPCR examination was plotted for each sample. The Student *t* test was used for comparing the groups

## Data Availability

The datasets used and/or analyzed during the current study are available from the corresponding author on reasonable request.
